# Development of a key performance indicator set for perioperative red blood cell transfusion

**DOI:** 10.1016/j.bjao.2024.100372

**Published:** 2025-01-30

**Authors:** Akshay Shah, Hayley G. Evans, Antony J.R. Palmer, Alan M. MacDonald, Martha Belete, Linda von Neree, Michael M.F. Murphy, Simon J. Stanworth, Robbie Foy, for the NIHR Blood and Transplant Research Unit in Data Driven Transfusion Practice

**Affiliations:** 1 Nuffield Department of Clinical Neurosciences, University of Oxford, Oxford, UK; 2 Department of Anaesthesia, Hammersmith Hospital, Imperial College Healthcare NHS Trust, London, UK; 3 NIHR Blood and Transplant Research Unit in Data Driven Transfusion Practice, Radcliffe Department of Medicine, University of Oxford, Oxford, UK; 4 Nuffield Department of Orthopaedics, Rheumatology and Musculoskeletal Sciences, University of Oxford, UK; 5 Nuffield Orthopaedic Centre, Oxford University Hospitals NHS Foundation Trust, Oxford, UK; 6 Poole Hospital, University Hospitals Dorset NHS Foundation Trust, Poole, UK; 7 Department of Anaesthesia, Torbay and South Devon NHS Foundation Trust, Torquay, UK; 8 Research and Audit Federation of Trainees, London, UK; 9 NIHR UCL Hospitals Biomedical Research Centre, London, UK; 10 Leeds Institute of Health Sciences, University of Leeds, Leeds, UK

**Keywords:** consensus, outcome performance, quality improvement, transfusion

## Abstract

**Background:**

Perioperative red blood cell (RBC) transfusion is a common intervention in patients undergoing surgery but there is marked variation in practice. Key performance indicators (KPIs) are central to identifying deviation from agreed standards and improving clinical outcomes. We aimed to identify KPIs which can potentially be measured from routinely collected electronic healthcare records.

**Methods:**

We undertook a three-stage process. First, we completed a scoping review to identify potential KPIs from relevant literature and clinical guidelines. Next, we conducted a modified RAND consensus process with a multidisciplinary panel including medical professionals, patients and public involvement members. The consensus panel rated these KPIs according to importance and feasibility.

**Results:**

We identified 28 candidate KPIs covering the entire perioperative RBC transfusion process. The majority of the KPIs focused on improving patient care around the time of decision to transfuse RBCs and transfusion safety. Clinical outcome KPIs included hospital length of stay, hospital acquired infection, mortality, and hospital readmission at 30 and 90 days. Five candidate KPIs were judged as unimportant whilst there were concerns around the feasibility of measurement using routine data for 14 candidate KPIs. The panel identified nine potential KPIs for future testing.

**Conclusions:**

Using a systematic, stepwise, transparent approach, we have identified a set of 28 KPIs for assessment, monitoring, and improvement of perioperative RBC transfusion. Future research is needed to further validate this set for external use and benchmarking between hospitals and departments.

Over the past decade, the evidence base in perioperative transfusion medicine has expanded, mainly because of findings from large randomised controlled trials,^1^ and initiatives such as patient blood management (PBM)^2^ and national haemovigilance systems. These publications have informed guidance on perioperative red blood cell (RBC) transfusion thresholds,^3^ interventions to minimise blood loss and reduce the risk of requiring a RBC transfusion,^4^^,^^5^ and efforts to improve transfusion safety. However, it is widely recognised that the translation of evidence and associated guidance into clinical practice is slow and unpredictable.^6^

RBC transfusions are a common intervention in the perioperative period. More than 1.6 million units of RBCs were issued in the United Kingdom (UK) in 2022,^7^ of which one-third were given to surgical patients.^8^ International estimates report 24–44% of all RBC units issued by blood services are for surgical inpatients.^9^^,^^10^ Although life saving, RBC transfusions are a finite resource associated with a broad range of transfusion-related adverse events,^7^^,^^11^ immunomodulation,^12^ and incompatible transfusion as a result of human error. In addition, observational data support an association between RBC transfusion and short- and long-term mortality in patients undergoing major elective abdominal surgery.^13^ There is also evidence of variation in perioperative RBC transfusion for certain types of major surgery associated with a high risk of requiring a RBC transfusion, in particular, cardiac surgery, vascular surgery, major cancer surgery, and complex orthopaedic surgery.^14^ Although a certain degree of variation is expected based on risk-adjusted case-mix, wide variation that cannot be explained by illness severity or patient preference likely reflects unwarranted variation in clinical practice.^15^^,^^16^ It is plausible that the adverse effects of RBC transfusion may be exacerbated by non-compliance with guidance.^3^

Measuring adherence to recommended or best practice is essential for identifying unwarranted variation, targeting improvement measures, and monitoring their impact to ultimately improve quality of care. Key performance indicators (KPIs) can be broadly defined as validated measures of care or explicit and measurable items which act as a building block in the assessment of care.^17^ As a first step towards addressing unwarranted variation in perioperative RBC transfusion, we aimed to identify and potentially develop a set of evidence-based KPIs with the greatest potential for improving quality of care, and which could be measured using routinely collected electronic healthcare data. The use of routinely collected data offers several advantages such as lower burden than manual paper-based data collection (and therefore improved efficiency and lower costs), more comprehensive and consistent data collection to allow comparisons between different hospitals, less risk of selection bias, collection of long-term clinical outcomes, and scope for delivering rapid, real-time feedback to clinicians and stakeholders.^18^

## Methods

This study is reported in accordance with the Preferred Reporting Items for Systematic Reviews and Meta-Analyses extension for Scoping Reviews (PRISMA-ScR)^19^ and the Conducting and REporting DElphi Studies (CREDES) guidelines.^20^ Ethical approval was waived by the research governance, ethics and assurance team, University of Oxford. We conducted a three-stage consensus development process to identify KPIs primarily based on their clinical importance and feasibility of data extraction from routinely collected healthcare data.

### Stage 1: Scoping review

The study protocol for the scoping review was prospectively registered on the Open Science Framework (https://osf.io/jcunm/).

#### Information sources and searches

The following databases were searched from 2000 onwards for guidelines and audits by an information specialist (CD): MEDLINE (Ovid), PubMed (NLM, for non-Medline articles only), Embase (Ovid), Trip Medical Database (for guidelines only). Searches were restricted to English language only. Reference lists of studies deemed eligible for inclusion were scanned for other studies of relevance. The search strategy is available in Supplementary Material 1.

#### Eligibility criteria

We included studies according to the following criteria:

(i) Population: studies recruiting adult participants, undergoing major elective cardiac or noncardiac surgery, and requiring an RBC transfusion in the perioperative period.
(ii) Concept: articles that identify or propose one or more KPIs related to perioperative RBC transfusion.
(iii) Context: all articles (original research, guidelines, and quality standards) published from the year 2000 onwards, or in English, to ensure relevance to the current healthcare context and feasibility.

We excluded case studies, abstracts from conference proceedings, and non-English language studies. Major surgery was pragmatically defined as a procedure expected to last >2 hour or when blood loss may exceed 500 ml.^21^ However, we recognise that there is no agreed consensus on this.

#### Conceptual model and data charting process

**Fig 1:**
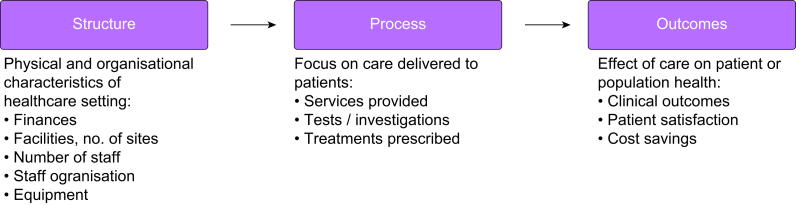
The Donabedian framework for quality of care.

We used the Donabedian conceptual framework for assessing quality of care using *structure, process*, and *outcome* components of quality, to map potential candidate KPIs and categorise them according to relevant thematic domains.^22^ In brief, this framework assumes a linear relationship where good structure measures drive good processes, which in turn leads to better outcomes (Fig. 1). We also reviewed and summarised current major international guidelines that include audit or quality improvement recommendations.

A pre-piloted data charting form was developed by the study team to determine which candidate indicators to extract. For the purposes of this review, the perioperative period was defined as the time around surgery and included three phases: preoperative, intraoperative, and postoperative. The identified KPIs were then categorised according to their relation to the transfusion process.

#### Data collection

Study characteristics that were extracted onto pre-piloted data extraction forms included, but were not limited to: publication type, study design, country, patient population (i.e. type of surgery), description of quality indicators including definition, selection and validation process (if any), numerator, denominator, and whether patients were involved in the development of the indicators.

### Stage 2: Indicator rating and selection

We convened a consensus panel comprising 12 members: four anaesthetists, three haematologists, three patient and public involvement (PPI) members with experience as blood donors or transfusion recipients, one surgeon, and one transfusion laboratory manager. Clinicians included a mix of early career and established researchers, all of whom are practicing in the UK. Supplementary Table S1 describes panel characteristics.

#### Virtual face-to-face consensus panel meeting

We used a modified RAND process, which is useful for judgements requiring deliberation and discussion.^23^ First, we conducted an online rating process (JISC Online surveys) whereby panellists rated each candidate KPI from Stage 1 according to two criteria: (i) importance (e.g. strength of supporting evidence, clinical importance, likelihood of cost savings without patient harm) and (ii) feasibility (e.g. amenability to measurement using routinely collected healthcare data). Panellists were instructed to rate all recommendations on a 9-point Likert scale (where 1 was low and 9 was high). We piloted this process with three clinicians and two lay people beforehand and responded by clarifying instructions and briefing each panellist individually before the rating process. PPI members rated each candidate KPI during an online meeting with the study team (6 September 2023) explaining each candidate KPI in lay terms and answering questions. PPI members were encouraged to rate each candidate KPI with an abstain option if they felt unable to do so. Before the consensus meeting, each panellist was individually informed of their own scores.

Panellists next attended a facilitated and structured online meeting (Zoom Video Communications Inc.; 21 September 2023). The study team presented median (range) scores for each candidate KPI and focused the discussion on those with maximal discordance, defined as at least two panellists scoring a candidate KPI 1–3 and at least two scoring it 7–9. Panellists had the opportunity to discuss the strength of the evidence for each candidate KPI, the importance of each candidate KPI to patients and the health service, clarify wording around some of the candidate KPIs, and discuss reasons for low or high ranking candidate KPIs.

After the meeting, panellists were encouraged to rate each candidate KPI again through an online rating process. They were advised to consider panel discussions and their own and aggregate initial scores. We aimed to take forward all the candidate KPIs rated as important and feasible, based on the level of agreement and median ratings from the rating process before and after the consensus panel meeting.

### Stage 3: Sense-checking and further refinement

We added this stage based on previous experience^24^ where certain KPIs may lack face validity as consensus panels overestimate the ability of using routinely collected data.^25^ We also wanted to ensure that identified KPIs were likely to be consistent with national guidelines^26^ and priorities whilst their measurement was unlikely to face ceiling effects. We therefore reviewed and, where necessary, refined or removed candidate indicators after research team discussions. Given that some promising candidate indicators were likely to be under-developed (e.g. lacking clearly defined denominator populations), we then elaborated potential numerators and denominators for each selected indicator.

Statistical analyses were undertaken using Microsoft Excel (Redmond, WA, USA) and Stata version 15.1 (STATACorp LP, College Station, TX, USA).

## Results

This study was conducted between May 2022 and December 2023.

### Stage 1: Scoping review

**Fig 2:**
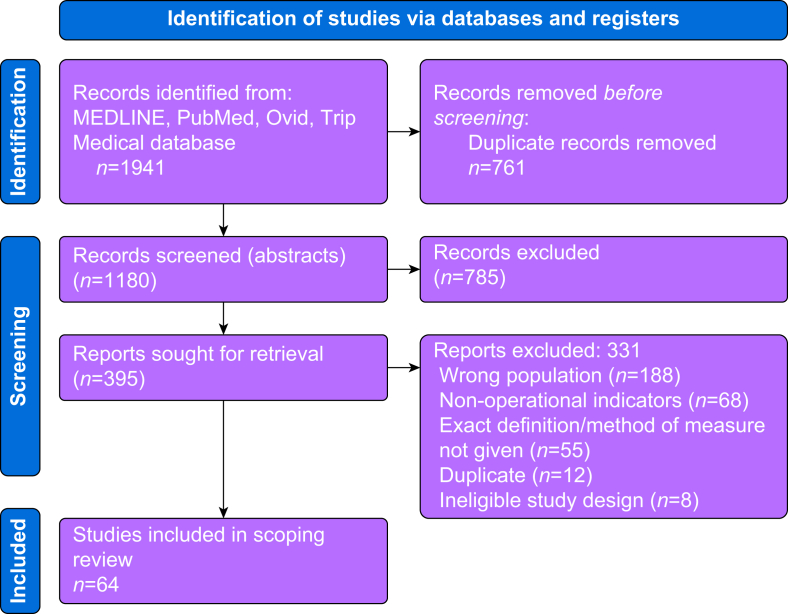
Preferred Reporting Items for Systematic Reviews and Meta-Analyses extension for Scoping Reviews (PRISMA) flow diagram describing study selection process.

The search retrieved 1941 references, reduced to 1180 once duplicates and clearly irrelevant references had been removed. From these 1180 references, 64 articles were eligible for inclusion (Fig. 2). Forty-four articles were primary research or quality improvement studies, and 20 articles were clinical guidelines. Characteristics of included studies are displayed in Table 1 and Supplementary Material 2. The majority of studies were conducted in patients undergoing orthopaedic or cardiac surgery. Half of all included studies used routine healthcare records as the primary data source. Twenty KPI recommendations for clinical guidelines are shown in Supplementary Table S2. Of these 20 guidelines, only eight recommended suggested KPIs and out of these only five provided further information on how to measure or operationalise the suggested KPI. From the 64 articles, 28 unique KPIs were identified and classified according to the Donabedian conceptual framework for assessing quality of care using *structure*, *process*, and *outcome* components of quality,^22^ and then by phase of transfusion (pre-transfusion, transfusion, post-transfusion) (Table 2).

**Table 1:** Characteristics of included studies^a^ (*n*=44).

Characteristic	*n* (%)
Study design	
- Audit/Quality Improvement	26 (59.1)
- Retrospective cohort	11 (25.0)
- Prospective cohort	2 (4.5)
- Mixed methods	3 (6.9)
- Survey	2 (4.5)
Surgical specialty	
- Orthopaedic	16 (36.4)
- Cardiac	11 (25.0)
- Major noncardiac:	17 (38.6)
Unspecified	12
Vascular	2
Oesophagectomy	1
Liver transplant	1
Major cancer	1
Geographical region	
- United Kingdom	13
- United States of America	10
- Asia	6
- Europe	7
- Australia	5
- South Africa	1
-Cross-continent (Australia, Europe)	1
- Unclear/not reported	1
Source of data collection	
- Routine healthcare records	22
- Manual data extraction	12
- Unclear/not reported	10
Method of data collection	
- Electronic	22
- Paper-based	4
- Bothelectronic and paper-based	2
- Unclear/not reported	16

QI, quality improvement.

a Excluded clinical guidelines.

**Table 2:** Candidate key performance indicators for perioperative RBC transfusion classified according to the Donabedian framework. AF, atrial fibrillation; C:T, crossmatch:transfusion ratio; G&S, group and screen; Hb, haemoglobin; LOS, length of stay; PBM, patient blood management; RBC, red blood cell; RRT, renal replacement therapy.

Category	Structure (facilities, equipment, resources, governance, training)	Processes (safety measures and timeliness, patient and user experience, ‘sum of all actions that make up healthcare’)	Outcomes (changes in health status/behaviours, mortality, quality of recovery)
Pre-transfusion	Use of computerised decision system Use of point-of-care testing Presence of hospital PBM committee Staff education programmes Utility and availability of cell salvage	Transfusion thresholds recommended by local/national guidelines (various) Sample labelling errors on tubes Preoperative screening and treatment of iron deficiency and anaemia at least 28 days before major surgery Measurement of Hb before each RBC transfusion	Prevalence of anaemia at (i) listing for surgery and (ii) on day of surgery Cancellation on day of surgery for anaemia Proportion of patients transfused after routine G&S Quality of red cell recovery from cell salvage (measured potassium, albumin, calcium) C:T ratio <2.5=significant blood utilisation
Transfusion	Presence/absence of availability of RBCs in theatre	Arrival of blood within 30 min for emergency blood Proportion of single-unit transfusions in stable, non-bleeding patients Average number of red cell units transfused per surgical procedure Massive transfusion, major haemorrhage, or both	Requirement for intraoperative transfusion Average number of red cell units transfused per surgical procedure
Post-transfusion	Reports provided back to staff members regarding their transfusion practice	Blood wastage Incidence of delayed transfusion defined as ‘>12 hours between the time at which threshold was reached and actual transfusion’ Appropriate documentation in medical records of blood loss, blood products transfused, and use of adjuncts (e.g. tranexamic acid, calcium)	Clinical/patient-centred: LOS, readmission rate (30, 60, and 90 days), mortality (in-hospital, 30 days) Postoperative complications—requirement for RRT, additional airway support, surgical site infection, AF, prolonged hospital stay (>7 days), hospital mortality Transfusion rates (% of patients transfused)

### Stage 2: Key performance indicator rating—importance and feasibility

**Fig 3:**
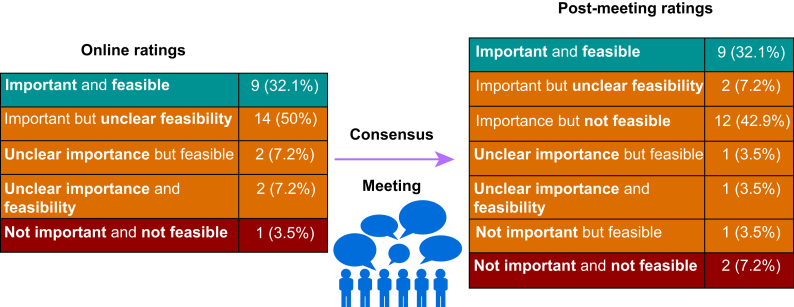
Changes in importance and feasibility of candidate key performance indicator (KPIs) during indicator selection process.

Online ratings across all 28 candidate KPIs were generally high for importance (mean ‘median’ score 7.48; standard deviation 0.84) with lower scores for feasibility (mean ‘median’ score 6.62; standard deviation 1.20) (Supplementary Table S3). Half of all candidate KPIs (*n*=14) were rated as being important but with uncertainty regarding feasibility of measurement using routinely collected data, whereas nine KPIs were rated as both important and feasible (Fig. 3). Only one candidate KPI was rated as being both unimportant and unfeasible by our consensus panel.

After the online consensus panel meeting, overall scores for importance (mean ‘median’ score 7.5; standard deviation 0.73) and feasibility were largely unchanged (mean ‘median’ score 6.71, standard deviation 1.02). Two candidate KPIs changed from having unclear importance to being unimportant. Fourteen candidate KPIs changed from having unclear feasibility or being feasible to being unfeasible (Fig. 3, Supplementary Table S4). Two candidate KPIs were now rated as being both unimportant and unfeasible, which included the one from the initial rating process.

### Stage 3: Sense-checking

Comments from Stage 2 raised concerns about difficulties in measurement of candidate KPIs from routinely collected data, disagreements between service level and clinical care indicators, and the supporting evidence base. Two examples included:

- Use of point-of-care testing devices (e.g. thromboelastography/rotational thromboelastometry)—this was judged to be a service level indicator and not an indicator with individual patient care as the unit of measurement. Their use has not yet been clearly associated with improvements in important clinical outcomes and the overall evidence is limited.^27^ In addition, there were also doubts about the feasibility of collecting these data from routinely available data.
- Postoperative complications—many complications were listed but the strength of the association with RBC transfusion was tenuous. Observational data support an association between RBC transfusion, venous thromboembolism^28^ and healthcare-acquired infection (HAI).^29^ Concerns were raised around feasibility of collecting viscoelastic testing data from routine data. Healthcare-aquired infection (HAI) was combined with the KPI on clinical outcomes.

We included candidate KPIs on the diagnosis of preoperative iron deficiency anaemia and use of tranexamic acid for surgery expected to have moderate blood loss because of their clinical importance^30^ and requirement for meeting national standards.^31^

By the end of this process, we had identified a list of nine KPIs which may serve as a starting point for future research. Seven of these KPIs were rated as both important and feasible by our panel (Table 3). These mainly covered avoiding the need for RBC transfusion where possible, minimising wastage, and the potential impact on patient-centred outcomes. Example numerator and denominator data, and data sources, which can be used to report events/numbers per 10 000 transfusions, are shown in Table 3.

**Table 3:** Suggested numerators and denominators for nine KPIs deemed suitable for future testing. EHR, electronic health records; G&S, group and screen; HAI, healthcare-acquired infection; Hb, haemoglobin; HES, hospital episode statistics; IDA, iron deficiency anaemia; GIRFT, Get It Right First Time; MHP, major haemorrhage protocol; OPCS, Office of Population Censuses and Surveys; RBC, red blood cells; SSI, surgical site infection; TXA, tranexamic acid; XM, crossmatch. ∗Electronic issue the selection and issue of red cell units where compatibility is determined by the Laboratory Information Management System (LIMS) without serological testing (serological crossmatch) of donor cells against patient plasma.

Performance indicator	Numerator	Denominator	Source of data collection
Adherence to RBC transfusion thresholds recommended by national/international clinical guidelines in stable, non-bleeding surgical patients	Number of transfusions occurring when Hb>70 g L^−1^	Number of surgical patients receiving a perioperative RBC transfusion	EHR
Measurement of haemoglobin after each RBC transfusion episode in stable, non-bleeding surgical patients	Number of patients where the Hb concentration is checked after each RBC transfusion	Number of surgical patients receiving an RBC transfusion	EHR
Sampling and labelling errors on blood tubes sent to blood bank (e.g. G&S)	Number of G&S/XM samples rejected for mislabelled specimens	Total number of G&S/XM samples collected	EHR
Proportion of patients who are expected to have moderate blood loss (at least 500 ml) or guidelines recommend TXA who receive tranexamic acid	Number of patients who received tranexamic acid	Number of patients who are having surgery and expected to have moderate blood loss (>500 ml) or guidelines recommend TXA	EHR For surgical procedures—see OPCS codes on GIRFT website
Proportion of people who are diagnosed with iron deficiency anaemia before surgery	Number of patients with laboratory results of IDA before surgery Number of patients referred to preoperative anaemia service	All patients undergoing major surgery	EHR
Clinical outcomes—hospital length of stay, hospital acquired infection, and mortality and hospital readmission at 30 and 90 days	Number of surgical patients who are readmitted or die after surgery	All patients undergoing major surgery	EHR OPCS codes for HAI HES
Inefficient use of RBCs for surgical patients	Number of RBCs ordered but not used/returned to blood bank (excluding electronic issue stock)∗	Total number of RBCs issued for surgical patients	Blood bank
Blood transfusion rates associated with surgery	Number of patients undergoing surgery and requiring an RBC transfusion in the perioperative period	Number of patients undergoing major surgery	EHR Blood bank
Proportion of surgical patients requiring a massive transfusion (>4 RBC units in 3 h or >10 RBC units in 24 h), activation of the major haemorrhage protocol, or both	Number of adults undergoing surgery and requiring >4 RBC units in 3 h or >10 RBC units in 24 h or activation of the MHP in the perioperative period	Number of adults undergoing major surgery	EHR Blood bank

## Discussion

After a scoping review and multidisciplinary consensus process, we identified 28 potential KPIs for perioperative RBC transfusion which can potentially be measured using routinely collected data in UK secondary care. These were almost evenly spread out across the structure, process, and outcome domains and covered the entirety of the perioperative RBC transfusion pathway.

**Fig 4:**
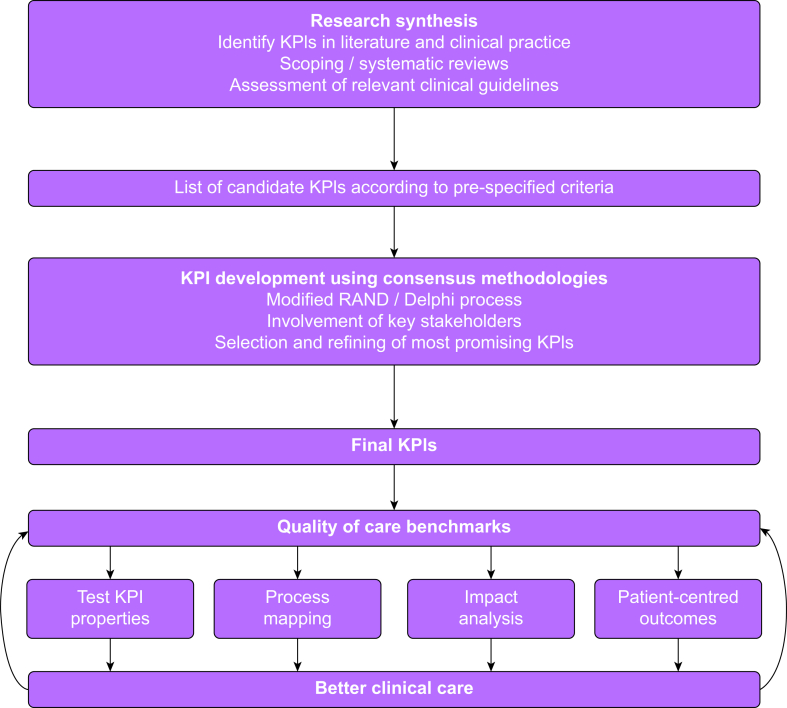
Overview of the key performance indicator development process. KPI, key performance indicator. (Adapted from Stelfox and Straus, *J Clin Epidemiol* 2013; **66**: 1328–37).^32^

Out of these, nine were deemed suitable as a starting point for further testing and validation. To our knowledge, this is the first set of KPIs developed for patients undergoing major surgery who may require a perioperative RBC transfusion. These indicators serve as a platform for field testing for feasibility of implementation and ability to reliably measure processes of care and improvements in associated outcomes (Fig. 4).

An ideal KPI should be: (i) based on agreed definitions; (ii) highly sensitive and specific; (iii) valid and reliable; (iv) able to discriminate well; (v) able to relate to clearly identifiable events for the user (e.g. patients, clinicians, managers); (vi) able to allow useful comparisons between hospitals; and (vii) evidence-based.^33^ One systematic review which identified 1282 perioperative structure and process indicators found that the majority of them (*n*=675 [53%]) did not have a level of evidence ascribed to them, and that only 13 indicators were judged to be patient-centric.^34^ Challenges to developing KPIs include difficulties in measurement (resource-intensive manual data extraction, overestimation of feasibility of data collection) and a lack of a supporting evidence. There may also be opportunity costs in investing time in measuring and attempting further improvement for indicators with likely ceiling effects, where adherence has reached a plateau beyond which it is difficult to improve practice further. During our Stage 2 process, 14 candidate KPIs changed from having unclear feasibility or being feasible to being unfeasible. However, a majority of candidate KPIs still remained important and future efforts should be targeted towards finding ways to measure these using routine healthcare data. Some candidate KPIs (e.g. presence of a hospital PBM committee, staff education programmes) were considered to be poor discriminators of quality.

The multidisciplinary nature of perioperative care means that identification of routine performance indicators or outcome metrics that are sensitive to variations in quality of care being delivered is challenging.^35^ Therefore, specific quality indicators have been described for monitoring particular aspects of care such as patient safety,^36^ regional anaesthesia,^37^ obstetric anaesthesia,^38^ and failure to rescue.^39^ Here, we describe a set of 28 KPIs to potentially allow hospitals to assess and improve perioperative RBC transfusion against agreed standards using routinely collected electronic healthcare data. The set developed is generic, and therefore potentially applicable to patients undergoing all types of surgery. An important consideration is to ensure widespread adoption and use of these KPIs. Various strategies have been described including integrated knowledge translation, where both the developers and end users of KPIs play a joint role in the entire development process and make KPIs easily accessible to end users.^32^ Alongside publications in journals, end user organisations (e.g. professional societies, healthcare providers) may post KPIs on their websites or incorporate them into any relevant clinical guidelines and quality standards. Relevant example organisations in the UK for our KPIs include the relevant Royal Colleges and NHS Blood and Transplant. Requirements for healthcare commissions to provide data on compliance with KPIs on a regular basis may also encourage their adoption and identify best practice. However, the dissemination of indicators is necessary but seldom sufficient by itself to bring about significant improvement. Active implementation strategies are usually necessary to change clinical practice, such as audit and feedback or clinical decision support.^40^ KPIs that draw upon routinely collected data can then be used both to help drive change (e.g. through performance feedback) and monitor progress.^41^

The utility of using electronic healthcare records was demonstrated in a clinical effectiveness and safety analysis of PBM in >1.2 million patients in Germany undergoing surgery.^2^ This study also demonstrated a time-dependent decay in PBM implementation. A more recent study in patients undergoing total hip or knee arthroplasty, from 33 hospitals in Spain, found suboptimal adherence to guideline-recommended PBM interventions with considerable variation between hospitals.^42^ Various strategies have been proposed to mitigate against this including clear accountability of roles and responsibilities, availability of strong leadership and champions, and provision of adequate support at an organisational level.^43^ In a complex intervention such as PBM, KPIs can have an important role in monitoring progress and make objective comparisons between different sites in order to enable a timely response to those who may be experiencing difficulties.

There are inevitable limitations to using electronic healthcare record systems, such as missing data and inadequate or absent coding, which are relevant to some of our KPIs. For measuring adherence to RBC transfusion thresholds recommended by guidelines, a pre-transfusion haemoglobin (Hb) may not always be available as laboratory workup is not mandatory for all surgical procedures. However, by focusing on major surgery only we expect that preoperative laboratory work and regular intraoperative monitoring will be part of standard care. Similarly, measurement of Hb after transfusion or when the patient is discharged from hospital may not always occur, but the absence of such measurement to monitor the outcome of transfusion may in itself be an important finding for sites to action. Point-of-care devices (e.g. HaemaCue) may also be used and these should be configured to transfer data to the main electronic record. Another potentially challenging KPI is identifying the proportion of patients with preoperative iron deficiency anaemia. Definitions of iron deficiency anaemia may vary between sites, specialties, and countries and therefore we have suggested a ‘surrogate’ measure such as referral to a preoperative anaemia clinic which can be extracted from electronic healthcare records.

The majority of our KPIs focused on measures to improve patient care around the time of decision to transfuse RBCs and transfusion safety (e.g. adherence to transfusion guidelines, measurement of Hb, sampling and labelling errors, inefficient use of RBCs) and on reducing the need for RBC transfusion (treating preoperative anaemia, use of tranexamic acid). The choice of clinical outcomes generated much discussion during our panel meeting. Patients value time spent at home and therefore it was unsurprising that length of stay, mortality, and readmission rates were consistently rated highly as being important and feasible. Many postoperative complications were considered as the PPI members felt that this is information they would want to know. However, collection of data on all complications possibly related to transfusion would have to be balanced against the likelihood of inclusion of many false positives. Hence, we agreed on measuring HAI because of its biologically plausible association with RBC transfusion. The proportion of patients requiring a massive transfusion was identified as an important KPI, especially in light of the 7th National Audit Project report which found that major haemorrhage was the commonest cause of perioperative cardiac arrest.^44^

Although our overall scores for each KPI did not change substantially after the consensus panel meeting, the exercise was still beneficial in helping us to identity 16 indicators with a perceived lack of feasibility. We have also provided a framework to operationalise these KPIs by providing suggested numerator and denominator data, and potential sources from where data may be obtained. Although we identified many guidelines on perioperative RBC transfusion, only eight recommended potential KPIs and of these only five provided methods on how to operationalise them. These observations may, in part, explain why implementation of guidelines is difficult to measure.

### Strengths and limitations

Strengths of our work include a systematic scoping review to inform the indicator selection process. Our panel was multidisciplinary, diverse, and included a broad range of clinicians, early career and established researchers, allied health professions and three PPI members. PPI during KPI development is very uncommon.^45^ However, our PPI members included one blood donor and two people with haematological conditions requiring multiple transfusions, thus representing the entire spectrum of the transfusion process from a PPI perspective. PPI members felt strongly about including efforts to minimise wastage of RBCs, provided input on choice of clinical outcomes that are important to patients and may be linked to RBC transfusion, and clarity of description of KPIs. Whilst there is a focus on treating iron deficiency anaemia, they also highlighted the important of not missing other disorders of iron homeostasis (e.g. iron overload).

We highlight four main study limitations. First, we have focused on identifying KPIs relevant for the UK secondary settings applicable to patients undergoing major surgery and receiving RBC transfusions. Other groups (e.g. obstetrics, cancer, critically ill) are also of importance and more work will be required to modify this list for those settings or other blood components. However, the evidence base and basic structures we have presented should be transferable to other, similar health services and clinical settings whilst our transparent methodology serves as a template for researchers to undertake such work. Second, our consensus panel meeting predominantly focused on KPIs where there was disagreement mainly to prioritise panellists' time on areas that would require higher degrees of judgement. Third, there is the potential that the use of a less formal sense-checking step could bring in bias. However, we felt the need to include this stage based on previous experience,^24^ suggesting that panellist meetings may not provide sufficient time to review all KPIs, and that expert panels developing KPIs can overestimate feasibility of data collection.^46^ Finally, there may be practical challenges in the actual measurement in practice of our KPIs (e.g. given limitations of clinical information systems).^41^ Nevertheless, we suggest our work is important in ensuring that the further development of information systems reflects clinical and administrative needs.

In summary, we have identified a set of 28 KPIs for assessment, monitoring, and improvement of perioperative RBC transfusion. From this set, nine KPIs were deemed to be potentially suitable for further testing and validation using routinely collected data in UK secondary care. These KPIs were developed with using a robust and transparent process which involved PPI members throughout.

## Author's contributions

Design and co-ordination of the study: AS, HGE, MMFM, AJRP, LVN SJS, RF

Scoping review: AS, HGE, AMM, MB

Modified RAND process: AS, RF, HGE, LVN, SJS

Data acquisition and analysis, AS, HGE, AJRP, SJS, RF

Involved in the interpretation of the data and the drafting of the manuscript: all authors

## Funding

The National Institute for Health and Care Research (NIHR) Blood and Transplant Research Unit in Data Driven Transfusion Practice (NIHR203334). The views expressed are those of the author(s) and not necessarily those of the NIHR or the Department of Health and Social Care.

## Declaration of interests

AS is an Editor of *Anaesthesia*.

## Appendix 1

NIHR BTRU in Data Driven Transfusion Practice Members

P. Dhiman https://orcid.org/0000-0002-0989-0623
L. Green https://orcid.org/0000-0003-4063-9768
A. Kotze https://orcid.org/0000-0002-9310-2895
S. E. Robinson https://orcid.org/0000-0001-5279-5536
F. Tomini https://orcid.org/0000-0003-2220-5210
S. Trompeter https://orcid.org/0000-0002-7099-8449
S. Warnakulasuriya https://orcid.org/0000-0001-8372-9857
W. K. Wong https://orcid.org/0000-0002-5742-0108
