# Supplementary material for: Development of a key performance indicator set for perioperative red blood cell transfusion
Source: BJA Open. 2025 Jan 30;13:100372. doi: 10.1016/j.bjao.2024.100372 (PMC11833354; doi:10.1016/j.bjao.2024.100372)
Supplement: Multimedia component 1 [file mmc1.docx]

**BTRU CLINICAL INDICATORS IN RED CELL TRANSFUSION**

**SEARCH NARRATIVE, APRIL 2022**

The following databases were searched from 2000 onwards for guidelines and audits on 4.4.22:

- MEDLINE (Ovid)
- PubMed (NLM, for non-Medline articles only)
- Embase (Ovid)
- Trip Medical Database (for guidelines only)

Searches were restricted to English language only and retrieved 1,941 references, which were reduced to 1,180 once duplicates and clearly irrelevant references had been removed. These were sent to the lead author, Simon Stanworth, in Word format on 5.4.22.

**SEARCH STRATEGIES**

**MEDLINE**

1. exp Clinical Audit/ or audit*.ti,bt,kf.

2. Quality Indicators, Health Care/ or (((clinical or quality or performance or guideline-based or evidence-based) adj (indicator* or metric*)) or clinical measure* or performance measure* or performance marker*).tw,kf.

3. (Guideline or Practice Guideline).pt. or (practice guideline* or clinical guideline*).ti,bt,kf.

4. 1 or 2 or 3

5. Erythrocyte Transfusion/ or Blood Transfusion/

6. (transfus* or PBM or blood product* or blood component* or blood management or blood therapy or exchange therapy or blood replacement).ti,bt,kf.

7. (blood adj3 (use* or usage* or utili* or administ* or need*)).ti,bt,kf.

8. (cell salvage or cell saver* or blood salvage or autotransfus* or auto transfus* or autohemotransfus* or auto haemotransfus*).ti,bt,kf.

9. exp Blood Group Incompatibility/

10. (mistransfus* or overtransfus* or mis-transfus* or over-transfus* or hemovigilance or haemovigilance).tw,kf.

11. (blood adj3 (contaminat* or infect* or incompatib* or mismatched)).ti,bt,kf.

12. (TRALI or TACO or TA GVHD or TAGVHD).ti,kf.

13. (transfus* adj3 (transmitt* or related or associated or trigger* or threshold*)).ab,kf.

14. (trauma* or polytrauma* or massive* haemorrhag* or massive* hemorrhag* or major bleed* or severe* bleed*).ti. and transfus*.ab.

15. ((blood adj3 cross match*) or (blood adj3 compatib*) or (blood adj3 incompatib*) or blood group* or (blood adj3 time limit*) or right blood or wrong blood or correct blood or incorrect blood or (blood adj3 storage) or stored blood or (blood adj3 collecti*) or (blood adj3 preserv*) or (blood adj3 conserv*)).ti,bt.

16. ((blood adj3 screen*) or (blood adj3 safe*) or (blood adj2 expos*) or (blood and virus transmission)).ti,bt.

17. (leukoreduc* or leucoreduc* or leukodeplet* or leucodeplet* or alloimmuni?ation).ti.

18. ((irradiat* or radiat* or nonirradiat* or preirradiat*) adj2 (blood or RBC* or red cell* or red blood cell*)).tw. and (transfus* or retransfus* or posttransfus* or post-transfus*).ti,bt,kf.

19. ((blood adj3 shortage*) or (blood adj2 exposure) or (blood adj2 storage) or (age adj2 blood) or (new* adj2 blood) or (old* adj2 blood) or (fresh* adj2 blood) or (store* adj2 blood) or (wast* adj3 blood)).ti,bt,kf.

20. or/5-19

21. 4 and 20

22. limit 21 to (english language and yr="2000 -Current")

**PubMed**

(transfus*[TI] OR RBC[TI] OR RBCs[TI] OR "red blood cells"[TI] OR "red cells"[TI] OR PBM[TI] OR "blood product"[TI] OR "blood products"[TI] OR "blood component"[TI] OR "blood components"[TI] OR "blood management"[TI] OR "blood therapy"[TI] OR "blood replacement"[TI]) AND (guideline[TI] OR audit*[TI] OR indicator*[TI] OR metric*[TI] OR measure*[TI] OR marker*[TI]) NOT medline[sb]

**Embase**

1. exp Clinical Audit/ or audit*.ti,bt,kf.

2. Clinical Indicator/ or (((clinical or quality or performance or guideline-based or evidence-based) adj (indicator* or metric*)) or clinical measure* or performance measure* or performance marker*).tw,kf.

3. *Practice Guideline/ or (practice guideline* or clinical guideline*).ti,bt,kf.

4. 1 or 2 or 3

5. *Blood Transfusion/ or Erythrocyte Transfusion/

6. Blood Transfusion Reaction/ or Transfusion Associated Graft versus Host Disease/

7. Transfusion Medicine/

8. (transfus* or PBM or blood product* or blood component* or blood management or blood therapy or exchange therapy or blood replacement).ti,bt,kf.

9. (blood adj3 (use* or usage* or utili* or administ* or need*)).ti,bt,kf.

10. (blood management or PBM or blood therapy or exchange therapy or blood replacement therapy).ti,bt,kf.

11. (cell salvage or cell saver* or blood salvage or autotransfus* or auto transfus* or autohemotransfus* or auto haemotransfus*).ti,bt,kf.

12. exp Blood Group Incompatibility/

13. (mistransfus* or overtransfus* or mis-transfus* or over-transfus* or hemovigilance or haemovigilance).tw,kf.

14. (blood adj3 (contaminat* or infect* or incompatib* or mismatched)).ti,bt,kf.

15. (TRALI or TACO or TA GVHD or TAGVHD).ti,kf.

16. (transfus* adj3 (transmitt* or related or associated or trigger* or threshold*)).ab,kf.

17. (trauma* or polytrauma* or massive* haemorrhag* or massive* hemorrhag* or major bleed* or severe* bleed*).ti. and transfus*.ab.

18. ((blood adj3 cross match*) or (blood adj3 compatib*) or (blood adj3 incompatib*) or blood group* or (blood adj3 time limit*) or right blood or wrong blood or correct blood or incorrect blood or (blood adj3 storage) or stored blood or (blood adj3 collecti*) or (blood adj3 preserv*) or (blood adj3 conserv*)).ti,bt.

19. ((blood adj3 screen*) or (blood adj3 safe*) or (blood adj2 expos*) or (blood and virus transmission)).ti,bt.

20. (leukoreduc* or leucoreduc* or leukodeplet* or leucodeplet* or alloimmuni?ation).ti.

21. ((irradiat* or radiat* or nonirradiat* or preirradiat*) adj2 (blood or RBC* or red blood cell* or red cell*)).tw. and (transfus* or retransfus* or posttransfus* or post-transfus*).ti,bt,kf.

22. ((blood adj3 shortage*) or (blood adj2 exposure) or (blood adj2 storage) or (age adj2 blood) or (new* adj2 blood) or (old* adj2 blood) or (fresh* adj2 blood) or (store* adj2 blood) or (wast* adj3 blood)).ti,bt,kf.

23. or/5-22

24. 4 and 23

25. limit 24 to (english language and embase and yr="2000 -Current")

**Trip Database**

Any of the Words in Title:

transfusion OR transfusions OR transfused OR transfusing OR RBC OR RBCs OR "red cells" OR "red blood cells" OR blood OR "cell salvage" OR autotransfusion OR autotransfusions OR "patient blood management" OR PBM

2000, 2022 – Guidelines
