# Supplementary material for: Development of a key performance indicator set for perioperative red blood cell transfusion
Source: BJA Open. 2025 Jan 30;13:100372. doi: 10.1016/j.bjao.2024.100372 (PMC11833354; doi:10.1016/j.bjao.2024.100372)
Supplement: Multimedia component 3 [file mmc3.docx]

**Supplementary Table S1.** Demographics of consensus panel (n=12)

| **Characteristic** | **n** |
| --- | --- |
| Sex   - Male: Female | 8:4 |
| Ethnicity   - White/Caucasian - Asian or Asian British - Black or Black British | 8  2  2 |
| Specialty   - Anaesthesia - Haematology - Surgery - Patient and public partner - Health informatics* | 4  4  1  3  3 |
| Years of experience in/with healthcare   - <5 years - 6-15 years - 16-25 years - 26-30 years - >30 years | 1  3  4  2  2 |

*Panel member with addition experience, qualifications and/or leadership roles in health informatics

**Supplementary Table S2.** Results table of key performance indicators recommended by international guidelines and audit tools

| **Guideline** | **KPI** | **Details on measurement/operational processes** |
| --- | --- | --- |
| General | | |
| Recommendations from the International Consensus Conference on Anemia Management in Surgical Patients (ICCAMS) Shander et al. 2022 | Patients should be educated about the impact of anaemia  Patients should be educated about the relationship between anaemia and RBC transfusion and the impact of increased RBC transfusion  All patients with anaemia should be evaluated for the cause of anaemia  The optimal timeframe to begin treating preoperative anemia prior to surgery is as soon as possible after surgery is scheduled, ideally at least 3–4 weeks in advance.  The treatment of postoperative anaemia should begin before discharge |  |
| Guidelines for the Management of Anaemia in the Perioperative Pathway McNally et al. 2022 | Development of a clinical pathway for the management of perioperative anaemia    Metrics to measure process  Measure patient reported outcomes  Workforce development | Number/proportion of patients presenting for major surgery identified to have anaemia  Number/proportion of patients with anaemia who required an intervention (eg iron infusion, blood transfusion) preoperatively  Number/proportion of patients with anaemia who required an intervention (eg blood transfusion) intraoperatively  Number/proportion of patients with anaemia who have required an intervention (eg iron infusion, blood transfusion) postoperatively  Number/proportion of patients with anaemia who have had surgery postponed/cancelled  Number/proportion of patients with anaemia who are referred postoperatively for further follow-up or management of anaemia at discharge  Availability of hospital guideline for detection and management of anaemia applicable to the perioperative setting  Length of hospital stay of patients undergoing major surgery who have anaemia  Length of hospital stay of patients undergoing major surgery who have needed an intervention (eg blood transfusion) to treat their anaemia  30-day readmission in patients with anaemia undergoing surgery  Decisional regret  Satisfaction with shared decision making (e.g. using SDMQ9) HRQoL e.g. EQ-5D-5L Number / proportion of staff working in perioperative care settings who have completed training up to (and including) eLFH level 3 training or equivalent  Availability of a team to support/deliver perioperative management of anaemia |
| Patient Blood Management: Recommendations From the 2018 Frankfurt Consensus Conference. Mueller et al. 2018 | None reported |  |
| Practice Guidelines for Perioperative Blood Management: An Updated Report by the American Society of Anesthesiologists Task Force on Perioperative Blood Management* Apfelbaum at al. 2014 | None reported |  |
| Clinical standards for patient blood management and perioperative hemostasis and coagulation management. Position Paper of the Italian Society of Anesthesia, Analgesia, Resuscitation and Intensive Care (SIAARTI)  Cinella et al 2019 | None reported |  |
| AAGBI guidelines: the use of blood components and their alternatives 2016.  Klein et al. 2016 | None reported |  |
| Association of Anaesthetists guidelines: cell salvage for peri-operative blood conservation 2018.  Klein et al. 2018 | Collection of blood for potential cell salvage (‘collect only’ mode) should be considered for surgical procedures where blood loss may exceed 500 ml (or > 10% of calculated total blood volume) in adult patients, or > 8 ml.kg^−1^ (> 10% of calculated total blood volume) in children weighing > 10 kg | Whenever CS is used, this should be accurately clinically coded to meet the approved NHS Fundamental Information Standard (SNOMED-CT code 233568002). This will enable appropriate reimbursement of the service and improve data collection and audit. |
| Korean clinical practice guideline for perioperative red blood cell transfusion from Korean Society of Anesthesiologists.  Koo et al. 2019 | None reported |  |
| British Committee for Standards in Haematology Guidelines on the Identification and Management of Pre-Operative Anaemia. Kotze et al. 2015 | All POAC’s should have a comprehensive written policy in place covering all aspects for the recognition, management and treatment of anaemia.  All patients who are identified as at risk of requiring a blood transfusion (according to local MSBOS – Group and save or cross-match) should have FBC assessed at POAC. Patients should also be given information about the possibility of requiring a blood transfusion, and, when appropriate, alternatives to transfusion e.g. intra-operative cell salvage  All FBC results should be reviewed within 2 working days  Those patients who were found to be anaemic should be re-assessed prior to listing for theatre.  POACs should audit their effectiveness of managing patients with anaemia and should be represented at the Hospital Transfusion Committee. |  |
| Management of severe perioperative bleeding: Guidelines from the European Society of Anaesthesiology.  Kozek-Langenecker et al. 2013 | None reported |  |
| Recommendations for the transfusion management of patients in the peri-operative period. I. The pre-operative period.; II. The intra-operative period;. III. The post-operative period.  Liumbruno et al. 2015 | None reported |  |
| Orthopaedics | | |
| Tranexamic Acid Use in Total Joint Arthroplasty: The Clinical Practice Guidelines Endorsed by the American Association of Hip and Knee Surgeons, American Society of Regional Anesthesia and Pain Medicine, American Academy of Orthopaedic Surgeons, Hip Society, and Knee Society. Fillingham et al. 2019 | None reported |  |
| Detection, evaluation, and management of preoperative anaemia in the elective orthopaedic surgical patient: NATA guidelines.  Goodnough et al. 2011 | None reported |  |
| Guideline for the management of hip fractures 2020: Guideline by the Association of Anaesthetists.  Griffiths et al. 2021 | Use of cell salvage and/or TXA in cases of expected blood loss >500 mls  Restrictive transfusion in the absence of blood loss, with a Hb target of 70 g/L in patients without cardiovascular risk factors  Clear plan for perioperative management of patients on anticoagulants, including bridging therapy and when to restart anticoagulants | % of eligible cases where TXA was used % of eligible cases where cell salvage was used  Documentation in notes of transfusion trigger  Audit frequency of single-unit transfusions compared with multiple in stable patients  Documentation of decision making Surveying awareness of pathways |
| Cardiac | | |
| 2017 EACTS/EACTA Guidelines on patient blood management for adult cardiac surgery Boer et al 2017 | None reported |  |
| The role of fibrinogen and fibrinogen concentrate in cardiac surgery: an international consensus statement from the Haemostasis and Transfusion Scientific Subcommittee of the European Association of Cardiothoracic Anaesthesiology.  Erdoes et al. 2019 | None reported |  |
| STS/SCA/AmSECT/SABM Update to the Clinical Practice Guidelines on Patient Blood Management.  Tibi et al. 2021 | None reported |  |
| Audit tools | | |
| Clinical Practice Guidelines From the AABB Red Blood Cell Transfusion Thresholds and Storage + AABB PBM White paper  Carson et al. 2016 | Overall transfusion rate compared with of similar size hospitals Transfusion rates for specific cases (orthopaedics, cardiac surgery) compared with national data % of transfusions that fall outside hospital/national guidelines Transfusion administration compliance Transfusion reaction rates Budget (inventory, supply costs, product) |  |
| NICE Guideline NG24 NICE 2015 | People should have their Hb checked at least 2 weeks before surgery, if possible and necessary for the procedure they are having. If they have iron deficiency anaemia, they should be offered iron supplementation. Oral iron should be offered initially and started at least 2 weeks before surgery. If oral iron is not appropriate, IV iron should be offered.  Adults who are having surgery and are expected to have moderate blood loss are offered TXA.  Reassessment after blood transfusions  Patient information | Does your hospital operate a pre-operative anaemia pathway?  How many patients received iron supplementation before surgery?  Does your hospital operate a post-operative anaemia pathway?  How many cases received TXA? OPCS procedure codes provided in toolkit for those expected to have moderate blood loss >500 mls  How many cases were clinically re-assessed after the red cell transfusion?  How many cases had Hb level checked after the red cell transfusion?  How many cases had BOTH a clinical re-assessment AND Hb level checked after the red cell transfusion?  How many of these cases have documented evidence that they were given verbal information about blood transfusion?  How many of these cases have documented evidence that they were given written information about blood transfusion?  How many of these cases have documented evidence that they were given verbal AND written information about blood transfusion? |
| RCoA Quality Improvement Compendium – Adherence to PBM in cardiac surgery,  RCoA 2020 | Preoptimisation of Hb    Use of antifibrinolytics  Use of red-cell salvage using centrifugation  Use of transfusion algorithm supplemented with point-of-care testing | Numbers of patients presenting for surgery with suboptimal haemoglobin  Numbers of patients with anaemia who had received supplemental iron (intravenously or orally)  Percentage of total patients with anaemia.  Percentage of patients who received intraoperative tranexamic acid or aprotinin.  Percentage of patients receiving cell-saved blood Volume of cell saver collected and given (do not include autologous, allogeneic, pump-residual, or chest-tube recirculated blood)  Percentage of patients receiving cardiotomy blood after bypass.  Does the unit use point-of-care testing? Presence of an evidence-based transfusion algorithm?  Percentage of patients having product transfusion without point-of-care-testing |

**Supplementary Table S3.** Scores of each candidate indicator pre- and post-consensus panel meeting

|  | **Round 1 Importance** | | | **Round 1 Feasibility** | | | **Round 2 Importance** | | | **Round 2 Feasibility** | | |
| --- | --- | --- | --- | --- | --- | --- | --- | --- | --- | --- | --- | --- |
| **Code** | **Mean** | **Median** | **Range** | **Mean** | **Median** | **Range** | **Mean** | **Median** | **Range** | **Mean** | **Median** | **Range** |
| kpi_1 | 7 | 8 | 3-8 | 7.5 | 8 | 6-9 | 7.1 | 8 | 5-8 | 7.4 | 8.0 | 5-8 |
| kpi_2 | 7.7 | 8 | 5-9 | 6.2 | 6 | 3-7 | 8.1 | 8 | 4-8 | 6.0 | 6.0 | 4-8 |
| kpi_3 | 7.3 | 7.5 | 5-9 | 7.4 | 8 | 6-9 | 7.8 | 8 | 7-9 | 8.1 | 8.0 | 7-9 |
| kpi_4 | 8.5 | 9 | 5-9 | 6.8 | 7.5 | 3-9 | 8.2 | 9 | 3-9 | 7.4 | 8.0 | 3-9 |
| kpi_5 | 6.2 | 6 | 4-9 | 6.9 | 8 | 2-9 | 6.2 | 6 | 5-9 | 7.8 | 8.0 | 5-9 |
| kpi_6 | 7.1 | 7 | 5-9 | 5.8 | 5.5 | 2-9 | 7.1 | 7 | 4-8 | 5.5 | 5.0 | 4-8 |
| kpi_7 | 7.8 | 8 | 5-9 | 5.9 | 5 | 3-9 | 7.8 | 8 | 4-9 | 6.5 | 6.0 | 4-9 |
| kpi_8 | 7 | 7 | 5-9 | 7.1 | 7.5 | 5-9 | 7.2 | 7 | 4-9 | 7.1 | 7.5 | 4-9 |
| kpi_9 | 6.6 | 7 | 3-9 | 6.0 | 6 | 2-9 | 6.9 | 7 | 3-9 | 6.4 | 6.5 | 3-9 |
| kpi_10 | 7.3 | 8 | 3-9 | 5.6 | 5.5 | 3-9 | 7.4 | 8 | 3-8 | 5.0 | 5.0 | 3-8 |
| kpi_11 | 6.8 | 7 | 5-9 | 6.8 | 7.5 | 2-9 | 7.3 | 8 | 2-9 | 6.5 | 7.0 | 2-9 |
| kpi_12 | 7.3 | 7.5 | 3-9 | 6.5 | 6.5 | 3-9 | 7.4 | 8 | 3-9 | 6.5 | 7.0 | 3-9 |
| kpi_13 | 8.3 | 8.5 | 7-9 | 6.1 | 7 | 1-9 | 8.0 | 8 | 3-9 | 6.5 | 6.5 | 3-9 |
| kpi_14 | 8 | 9 | 3-9 | 5.1 | 6 | 1-9 | 7.6 | 8 | 3-9 | 5.6 | 5.5 | 3-9 |
| kpi_15 | 6.4 | 6 | 3-9 | 6.3 | 7 | 2-9 | 6.4 | 7 | 2-9 | 5.6 | 6.0 | 2-9 |
| kpi_16 | 6.6 | 6.5 | 1-9 | 6.4 | 7 | 3-9 | 6.3 | 6.5 | 6-9 | 7.8 | 8.0 | 6-9 |
| kpi_17 | 7 | 7.5 | 4-9 | 6.6 | 6 | 5-9 | 7.0 | 7 | 6-9 | 7.0 | 6.5 | 6-9 |
| kpi_18 | 6.6 | 7 | 3-9 | 5.8 | 6.5 | 2-8 | 6.2 | 7 | 2-8 | 5.1 | 5.0 | 2-8 |
| kpi_19 | 6.2 | 6.5 | 2-9 | 4.3 | 3.5 | 1-9 | 6.5 | 6.5 | 1-8 | 5.1 | 6.0 | 1-8 |
| kpi_20 | 7.2 | 7 | 4-9 | 4.9 | 5 | 1-9 | 7.4 | 8 | 1-9 | 5.5 | 6.0 | 1-9 |
| kpi_21 | 8.2 | 8.5 | 6-9 | 7.6 | 8 | 4-9 | 8.2 | 8 | 4-9 | 7.4 | 8.0 | 4-9 |
| kpi_22 | 7.3 | 8 | 1-9 | 7.4 | 8 | 5-9 | 7.6 | 8 | 5-9 | 7.4 | 8.0 | 5-9 |
| kpi_23 | 5.7 | 6 | 1-9 | 6.1 | 6 | 3-9 | 6.1 | 6 | 3-9 | 6.4 | 6.0 | 3-9 |
| kpi_24 | 7.2 | 7 | 4-9 | 7.5 | 7.5 | 6-9 | 7.3 | 8 | 5-9 | 7.0 | 7.0 | 5-9 |
| kpi_25 | 7.8 | 8 | 5-9 | 6.4 | 6 | 4-9 | 7.9 | 8 | 2-9 | 6.3 | 6.0 | 2-9 |
| kpi_26 | 6.9 | 8 | 3-9 | 7.4 | 8 | 5-9 | 7.4 | 8 | 5-9 | 7.5 | 8.0 | 5-9 |
| kpi_27 | 7 | 8 | 3-9 | 5.7 | 5 | 3-9 | 7.2 | 7 | 3-9 | 5.9 | 6.5 | 3-9 |
| kpi_28 | 7.2 | 8 | 3-9 | 7.4 | 8 | 5-9 | 7.1 | 7 | 5-9 | 7.0 | 7.0 | 5-9 |
| Mean |  | 7.48 |  |  | 6.62 |  |  | 7.5 |  |  | 6.71 |  |
| SD |  | 0.84 |  |  | 1.20 |  |  | 0.73 |  |  | 1.02 |  |

**KPI codes**

| kpi_1: Adherence to transfusion thresholds recommended by local/national clinical guidelines |
| --- |
| kpi_2: Preoperative screening and treatment of iron deficiency anaemia at least 28 days before major surgery |
| kpi_3: Measurement of haemoglobin before each red blood cell transfusion |
| kpi_4: Sampling / labelling errors on blood tubes (e.g. Group and Save) |
| kpi_5: Presence of a hospital Patient Blood Management committee |
| kpi_6: Staff education programmes |
| kpi_7: Availability and utility of cell salvage |
| kpi_8: Use of point-of-care testing devices (e.g. TEG/ROTEM) |
| kpi_9: Use/presence of a computerised clinical decision support system |
| kpi_10: Cancellation on day of surgery for anaemia |
| kpi_11: Prevalence of anaemia at (i) listing for surgery; and/or (ii) on day of surgery |
| kpi_12: Availability of red blood cells in the theatre suite/complex |
| kpi_13: Arrival of emergency blood within 30 minutes of request |
| kpi_14: Appropriate documentation in medical records of blood loss, blood products transfused and use of adjuncts (e.g. calcium, tranexamic acid) |
| kpi_15: Incidence of delayed transfusion, defined as '>12 hours between the time at which transfusion threshold was reached and actual transfusion' |
| kpi_16: Proportion of single-unit red blood cell transfusions in stable, non-bleeding patients |
| kpi_17: Requirement for intra-operative red blood cell transfusion |
| kpi_18: Feedback process e.g. reports provided back to staff members/departments regarding their transfusion practice |
| kpi_19: Quality of red cell recovery from cell salvage (i.e. through measured potassium, albumin, calcium) |
| kpi_20: Postoperative blood loss in the first 12 hours following surgery |
| kpi_21: Clinical / patient-centered outcomes, in particular hospital length of stay, mortality and readmissions (within 30 and/or 90 days) |
| kpi_22: Wastage of red blood cell units |
| kpi_23: Cross match:transfusion ratio of >2.5 (as an indicator of substantial blood utilisation) |
| kpi_24: Average number of red blood cells transfused per surgical procedure |
| kpi_25: Postoperative complications, in particular surgical site infection, additional airway support, renal replacement therapy, thrombosis, prolonged hospital stay (>7 days) |
| kpi_26: Transfusion rates (as a percentage of patients transfused) |
| kpi_27: Measurement of haemoglobin at hospital discharge |
| kpi_28: Percentage of patients requiring a massive transfusion and/or activation of the major haemorrhage protocol |

**Supplementary Table S4**. Changes in importance and feasibility of candidate key performance indicators during indicator selection process

|  | Round 1 | | Round 2 | |
| --- | --- | --- | --- | --- |
| **KPI** | Importance | Feasibility | Importance | Feasibility |
| (1) Adherence to transfusion thresholds recommended by local/national clinical guidelines |  |  |  |  |
| (2) Preoperative screening and treatment of iron deficiency anaemia at least 28 days before major surgery |  |  |  |  |
| (3) Measurement of haemoglobin before each red blood cell transfusion |  |  |  |  |
| (4) Sampling / labelling errors on blood tubes (e.g. Group and Save) |  |  |  |  |
| (5) Presence of a hospital Patient Blood Management committee |  |  |  |  |
| (6) Staff education programmes |  |  |  |  |
| (7) Availability and utility of cell salvage |  |  |  |  |
| (8) Use of point-of-care testing devices (e.g. TEG/ROTEM) |  |  |  |  |
| (9) Use/presence of a computerised clinical decision support system |  |  |  |  |
| (10) Cancellation on day of surgery for anaemia |  |  |  |  |
| (11) Prevalence of anaemia at (i) listing for surgery; and/or (ii) on day of surgery |  |  |  |  |
| (12) Availability of red blood cells in the theatre suite/complex |  |  |  |  |
| (13) Arrival of emergency blood within 30 minutes of request |  |  |  |  |
| (14) Appropriate documentation in medical records of blood loss, blood products transfused and use of adjuncts (e.g. calcium, tranexamic acid) |  |  |  |  |
| (15) Incidence of delayed transfusion, defined as '>12 hours between the time at which transfusion threshold was reached and actual transfusion' |  |  |  |  |
| (16) Proportion of single-unit red blood cell transfusions in stable, non-bleeding patients |  |  |  |  |
| (17) Requirement for intra-operative red blood cell transfusion |  |  |  |  |
| (18) Feedback process e.g. reports provided back to staff members/departments regarding their transfusion practice |  |  |  |  |
| (19) Quality of red cell recovery from cell salvage (i.e. through measured potassium, albumin, calcium) |  |  |  |  |
| (20) Postoperative blood loss in the first 12 hours following surgery |  |  |  |  |
| (21) Clinical / patient-centered outcomes, in particular hospital length of stay, mortality and readmissions (within 30 and/or 90 days) |  |  |  |  |
| (22) Wastage of red blood cell units |  |  |  |  |
| (23) Cross match:transfusion ratio of >2.5 (as an indicator of substantial blood utilisation) |  |  |  |  |
| (24) Average number of red blood cells transfused per surgical procedure |  |  |  |  |
| (25) Postoperative complications, in particular surgical site infection, additional airway support, renal replacement therapy, thrombosis, prolonged hospital stay (>7 days) |  |  |  |  |
| (26) Transfusion rates (as a percentage of patients transfused) |  |  |  |  |
| (27) Measurement of haemoglobin at hospital discharge |  |  |  |  |
| (28) Percentage of patients requiring a massive transfusion and/or activation of the major haemorrhage protocol |  |  |  |  |
